# Modelling Adaptive Learning Behaviours for Consensus Formation in Human Societies

**DOI:** 10.1038/srep27626

**Published:** 2016-06-10

**Authors:** Chao Yu, Guozhen Tan, Hongtao Lv, Zhen Wang, Jun Meng, Jianye Hao, Fenghui Ren

**Affiliations:** 1 School of Computer Science and Technology, Dalian University of Technology, Dalian, 116024, Liaoning, China; 2 School of Software, Dalian University of Technology, Dalian, 116621, China; 3 School of Computer Engineering, Nanyang Technological University, 639798, Singapore; 4 School of software, Tianjin University, Tianjin, 300072, China; 5 School of Computer Science and Software Engineering, University of Wollongong, Wollongong, 2500, Australia

## Abstract

Learning is an important capability of humans and plays a vital role in human society for forming beliefs and opinions. In this paper, we investigate how learning affects the dynamics of opinion formation in social networks. A novel learning model is proposed, in which agents can dynamically adapt their learning behaviours in order to facilitate the formation of consensus among them, and thus establish a consistent social norm in the whole population more efficiently. In the model, agents adapt their opinions through trail-and-error interactions with others. By exploiting historical interaction experience, a guiding opinion, which is considered to be the most successful opinion in the neighbourhood, can be generated based on the principle of evolutionary game theory. Then, depending on the consistency between its own opinion and the guiding opinion, a focal agent can realize whether its opinion complies with the social norm (i.e., the majority opinion that has been adopted) in the population, and adapt its behaviours accordingly. The highlight of the model lies in that it captures the essential features of people’s adaptive learning behaviours during the evolution and formation of opinions. Experimental results show that the proposed model can facilitate the formation of consensus among agents, and some critical factors such as size of opinion space and network topology can have significant influences on opinion dynamics.

Opinion dynamics is an attempt at understanding the evolution and formation of social opinions achieved through microscopic interactions between individuals in a multiagent society^1^,^2^,^3^,^4^. Researchers from a variety of disciplines including statistical physics, econophysics, sociophysics and computer science have made significant contributions to this field^5^,^6^,^7^. By using theoretical models and experimental methods, socially macroscopic phenomena such as global consensus (i.e., social norm), polarization, or anarchy (diversity of opinions) can be observed and analyzed, providing us a comprehensive understanding of the dynamics of evolution and formation of opinions^8^,^9^,^10^, social conventions and rules^11^,^12^, as well as languages^13^,^14^ in human societies.

In the literature, a number of opinion dynamics models, such as the classic voter model^15^, the Galam model^16^, the social impact model^17^, the Sznajd model^18^, the Deffuant model^19^ and the Kraus-Hegselmanmodel model^20^, have been proposed and extensively analyzed. Other models have focused on investigating the influence of social factors such as information sharing or exchange on the evolution of opinions^21^,^22^. Also, there is abundant of research in the area of evolutionary game theory to investigate how opinions (i.e., defection and cooperation) evolve based on their interaction performance^23^. In most opinion dynamics models, each individual is considered to be an agent holding continuous or discrete opinions in favor of one decision or choice (accept/reject, or cooperate/defect), and each individual interacts with others and tries to persuade or impact others through his/her opinion. The focus is on investigating macroscopic phenomenon achieved through local dynamics that are based on simple social learning rules, such as local majority and conformity^8^,^24^, imitating a neighbor^23^,^25^, or the coupling of these two rules^26^,^27^.

In real-life situations, however, people’s decision making is far more complex than simple imitation or voting. Rather, people usually learn through trail-and-error interactions with others when facing uncertainties about their decisions or choices. This kind of experience-based learning is an essential capability of human and plays a vital role in human society for facilitating coordination and cooperation among individuals and thus sustaining global social order in the society^28^,^29^. In this sense, the observed macroscopic consistency of human behavior is essentially an outcome of a local learning process. Understanding how global consensus can be achieved through each individual’s local learning experience thus becomes a critical problem in the research of opinion dynamics.

In this paper, we try to investigate the impact of learning from local interactions on the dynamics of opinion formation in a population of networked agents. Specially, we focus on analysing how adaptive behaviors during learning can facilitate the establishment of global consensus among agents. In the model, each agent is associated with a number of discrete opinions and try to reach an agreement about their opinions through interactions with other agents in its neighbourhood. Each agent evaluates the effect of its expressed opinion based on the positive or negative outcome of the interaction with other agents and tries to choose the opinion with the best performance. This process can be realized through a reinforcement learning (RL) process^30^, which provides a general approach to model how an agent can achieve an optimal performance through trail-and-error interactions with its environment. The learning experience in terms of expressed opinion with its corresponding outcome is stored in a memory with certain length. The historical learning experience of each agent is then synthesised into a strategy that competes with other strategies in the neighbourhood. The strategy that has better performance is more likely to survive and thus be accepted by other agents as a guiding opinion to adapt their own opinions. This competing process can be carried out through a social learning process based on the principle of Evolutionary Game Theory (EGT)^23^,^25^, which provides a powerful methodology to model how strategies evolve overtime based on their performance. Based on the consistency between the agent’s chosen opinion and the guiding opinion, the agent can dynamically adapt its learning behavior (in terms of learning and/or exploration rate) using a simple heuristic of “Win-or-Learn-Fast”. In this way, agents’ learning behaviours can be dynamically adapted according to the varying situations during the process of opinion formation. Extensive experiment has been carried out to investigate the dynamics of consensus formation under the proposed model, compared against a static learning (denoted as SL thereafter) model proposed in^31^,^32^. In SL model, each agent interacts with one of its neighbours and adapts its opinion directly based on the outcome of that interaction. Comparing with this model thus enables to demonstrate the merits of the adaptive learning behavior of agents in influencing the consensus formation among agents. In order to provide a comprehensive verification of the proposed learning model, three evaluation criteria are considered. They are: (1) *Effectiveness* (i.e., possibility of achieving a consensus), denoting the percentage of runs in which a consensus can be successfully established; (2) *Efficiency* (i.e., convergence speed of achieving a consensus), indicating how many steps are needed for a consensus formation; and (3) *Efficacy* (i.e., level of consensus), indicating the ratio of agents in the population that can achieve the consensus. Note that, although the default meaning of *consensus* indicates that all the agents should have reached an agreement, we consider that the consensus can only be achieved at different levels in this paper. This is because achieving 100% consensus through local learning interactions is an extremely challenging issue due to the widely recognized existence of subnorms in the network, as reported in previous studies^12^,^28^. We consider three different kinds of topologies to represent an agent society. They are regular square lattice networks, small-world networks^33^ and scale-free networks^34^. Results show that the proposed model can facilitate the consensus formation among agents and some critical factors such as the size of opinion space and network topology can have significant influences on the dynamics of consensus formation among agents.

## Model

In the model, agents have *N*_*o*_ discrete opinions to choose from and try to coordinate their opinions through interactions with other agents in the neighbourhood. Initially, agents have no bias regarding which opinion they should choose. This means that the opinions are equally chosen by the agents at first. During each interaction, agent *i* and agent *j* choose opinion *o*_*i*_ and opinion *o*_*j*_ from their opinion space, respectively. If their opinions match each other (i.e., *o*_*i*_ = *o*_*j*_), they will get an immediate positive payoff of 1, and −1 otherwise. The payoff is then used as an appraisal to evaluate the expected reward of the opinion adopted by the agent, which can be realized through a reinforcement learning (RL) process^30^. There are a variety of RL algorithms in the literature, among which Q-learning^35^ is the most widely used one. In Q-learning, an agent makes a decision through estimation of a set of Q-values, which are updated by:


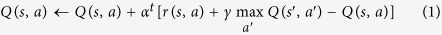


In Equation 1, *α*^*t*^ ∈ (0, 1] is learning rate of agent at step *t*, and *γ* ∈ [0, 1) is a discount factor, *r*(*s*, *a*) and *Q*(*s*, *a*) are the immediate and expected reward of choosing action *a* in state *s* at time step *t*, respectively, and Q(*s*′, *a*′) is the expected discounted reward of choosing action *a*′ in state *s*′ at time step *t* + 1. Q-values of each state-action pair are stored in a table for a discrete state-action space. At each time step, agent *i* chooses the best-response action with the highest Q-value based on the corresponding Q-values with a probability of 1 − *ε* (i.e., exploitation), or chooses other actions randomly with a probability of *ε* (i.e., exploration). In our model, action *a* in *Q*(*s*, *a*) represents the opinion adopted by the agent and the value of *Q*(*s*, *a*) represents the expected reward of choosing opinion *a*. As we do not model state transitions of agents, the stateless version of Q-learning is used. Thus, Equation 1 can be reduced to *Q*(*o*) ← *Q*(*o*) + *α*^*t*^[*r*(*o*) − *Q*(*o*)], where *Q*(*o*) is the Q-value of opinion *o*, and *r*(*o*) is the immediate reward of interaction using opinion *o*.


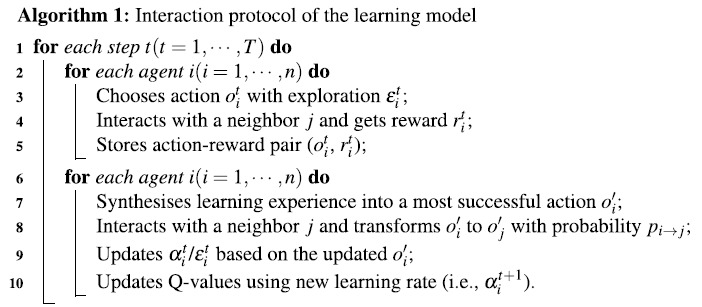


Based on Q-learning, interaction protocol under the proposed model (given by Algorithm 1) is briefly described as follows:

1. At each time step *t*, agent *i* chooses action (i.e., opinion) 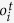 with the highest Q-value or randomly chooses an opinion with an exploration probability 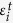 (Line 3). Agent *i* then interacts with a randomly selected neighbor *j* and receives a payoff of 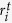 (Line 4). The learning experience in terms of action-reward pair 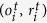 is then stored in a certain length of memory (Line 5);
2. The past learning experience (i.e., a list of action-reward pairs) contains the information of how often a certain opinion has been chosen and how this opinion performs in terms of its average reward achieved. Agent *i* then synthesises its learning experience into a most successful opinion 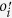 based on two proposed approaches (Line 7). This synthesising process will be described in detail in the following text. Agent *i* then interacts with one of its neighbours using 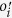, and generates a guiding opinion in terms of the most successful opinion in the neighbourhood based on the EGT (Line 8);
3. Based on the consistency between the agent’s chosen opinion and the guiding opinion, agent *i* adjusts its learning behaviours in terms of learning rate 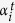 and/or the exploration rate 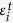 accordingly (Line 9);
4. Finally, agent *i* updates its Q-value using the new learning rate 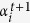 by Equation (1) (Line 10).

In this paper, the proposed model is simulated in a synchronous manner, which means that all the agents conduct the above interaction protocol concurrently.

Each agent is equipped with a capability to memorize a certain period of interaction experience in terms of the opinion expressed and the corresponding reward. Assuming a memory capability is well justified in social science, not only because it is more compliant with real scenarios (i.e., humans do have memories), but also because it can be helpful in solving challenging puzzles such as emergence of cooperative behaviours in social dilemmas^36^,^37^. Let *M* denote an agent’s memory length. At step *t*, the agent can memorize the historical information in the period of *M* steps prior to *t*. A memory table of agent *i* at time step *t*, 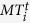, then can be denoted as 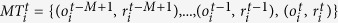. Based on the memory table, agent *i* then synthesises its past learning experience into two tables 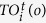 and 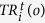. 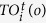 denotes the frequency of choosing opinion *o* in the last *M* steps and 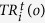 denotes the overall reward of choosing opinion *o* in the last *M* steps. Specifically, 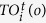 is given by:


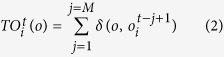


where 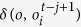 is the Kronecker delta function, which equals to 1 if 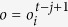, and 0 otherwise.

Table 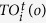 stores the historical information of how often opinion *o* has been chosen in the past. To exclude those actions that have never been chosen, a set *X*(*i*, *t*, *M*) is defined to contain all the opinions that have been taken at least once in the last *M* steps by agent *i*, i.e., 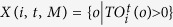. The average reward of choosing opinion *o*, 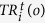, then can be given by:


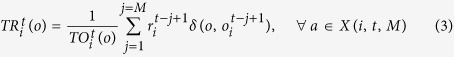


The past learning experience in terms of table 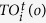 and 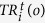 indicates how successful the strategy of choosing opinion *o* is in the past. This information is exploited by the agent in order to generate a guiding opinion. To realize the guiding opinion generation, each agent learns from other agents by comparing their learning experience. The motivation of this comparison comes from the EGT, which provides a powerful methodology to model how strategies evolve overtime based on their performance. In the context of EGT, an individual’s payoff represents its fitness or social success. The dynamics of strategy change in a population is governed by social learning, that is, the most successful agents will tend to be imitated by the others. Two different approaches are proposed in this model to realize the EGT concept, depending on how to define the competing strategy and the corresponding performance evaluation criteria (i.e., fitness) in EGT. They are performance-driven approach and behavior-driven approach, respectively:

- *Performance-driven approach*: This approach is inspired by the fact that agents are aiming at maximizing their own rewards. If an opinion has brought about the highest reward among all the opinions in the past, this opinion is the most profitable one and thus should be more likely to be imitated by the others in the population. Therefore, the strategy in EGT is represented by the most profitable opinion, and the fitness is represented by the corresponding reward of that opinion. Let 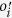 denote the most profitable opinion. It can be given by:

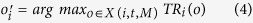
- *Behavior-driven approach*: In the behavior-driven approach, if an agent has chosen the same opinion all the time, it considers this opinion to be the most successful one (being the norm accepted by the population). Therefore, behavior-driven approach considers the opinion which has been most adopted in the past to be the strategy in EGT, and the corresponding reward of that opinion to be the fitness in EGT. Let 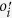 denote the most adopted opinion. It can be given by:

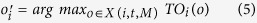
- After synthesising the historical learning experience, agent *i* then gets an opinion of 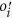 and its corresponding fitness of 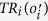. It then interacts with other agents through social learning based on the Proportional Imitation (PI)^23^ rule in EGT, which can be realized by the famous Fermi function:

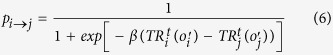

where *p*_*i*→*j*_ denotes the probability that agent *i* switches to the opinion of agent *j* (i.e., agent *i* remains opinion 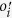 with a probability of 1 − *p*_*i*→*j*_), and *β* is a parameter to control the selection bias. Based on the principle of EGT, a guiding opinion represented as the new opinion 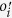 is generated. The new opinion 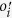 indicates the most successful opinion in the neighborhood and therefore should be integrated into the learning process in order to entrench its influence. By comparing its opinion at time step *t* (i.e., 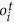) with the guiding opinion 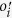, agent *i* can evaluate whether it is performing well or not so that its learning behavior can be dynamically adapted to fit the guiding opinion. Depending on the consistency between the agent’s opinion and the guiding opinion, the agent’s learning process can be adapted according to the following three mechanisms:
- SLR (Supervising Learning Rate *α*): In RL, the learning performance heavily depends on the learning rate parameter, which is difficult to tune. This mechanism adapts the learning rate *α* in the learning process. When agent *i* has chosen the same opinion with the guiding opinion, it decreases its learning rate to maintain its current state, otherwise, it increases its learning rate to learn faster from its interaction experience. Formally, learning rate 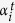 can be adjusted according to:

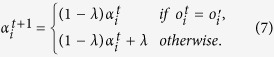

where *λ* ∈ [0, 1] is a parameter to control the adaption rate;
- SER (Supervising Exploration Rate *ε*): Exploration-exploitation trade-off has a crucial impact on the learning process. Therefore, this mechanism adapts the exploration rate *ε* in the learning process. The motivation of this mechanism is that an agent needs to explore more of the environment when it is performing poorly and explore less otherwise. Similarly, the exploration rate 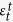 can be adjusted according to:

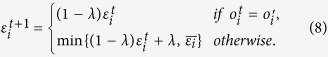

in which 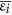 is a variable to confine the exploration rate to a small value in order to indicate a small probability of exploration in RL;
- SBR (Supervising Both Rates): This mechanism adapts the learning rate and the exploration rate at the same time based on SLR and SER. Learning rate and exploration rate are two fundamental tuning parameters in RL. Heuristic adaption of these two parameters thus models the adaptive learning behavior of agents. The proposed mechanisms are based on the concept of “winning” and “losing” in the well-known MAL algorithm WoLF (Win-or-Learn-Fast)^38^. Although the original meaning of “winning” or “losing” in WoLF and its variants is to indicate whether an agent is doing better or worse than its Nash-Equilibrium policy, this heuristic is gracefully introduced into the proposed model to evaluate the agent’s performance against the guiding opinion. Specifically, an agent is considered to be winning (i.e., performing well) if its opinion is the same with the guiding opinion and losing (i.e., performing poorly) otherwise. The different situations of “winning” or “losing” thus indicate whether the agent’s opinion is complying with the norm in the society. If an agent is in a losing state (i.e., its action is against the norm in the society), it needs to learn faster or explores more of the environment in order to escape from this adverse situation. On the contrary, it should decrease its learning and/or exploration rate to stay in the winning state.

## Results

**Figure 1:**
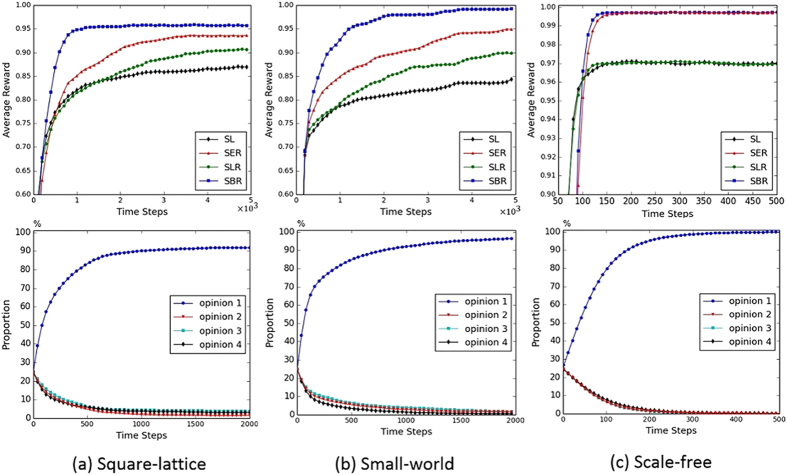
Dynamics of consensus formation in three different kinds of networks. The above is average reward of agents in the network and the bottom are the results of the frequency of agents’ opinions using approach SBR. Each agent has 4 opinions to choose from and a memory length of 4 steps. Behaviour-driven approach is used for the guiding opinion generation method. In the small-world network, *p* = 0.1 and *K* = 12. In Q-learning, *α* = 0.1, *ε* = 0.01, and 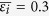. *β* in Equation 6 is 0.1 and *λ* in Equation 7 and 8 is 0.1. The agent population is 100 and the curves are averaged over 10000 Monte Carlo runs.

The dynamics of consensus formation in three different kinds of networks using static learning approach SL, and adaptive learning approaches SER, SLR and SBR are plotted in Fig. 1. The Watts-Strogatz model^33^ is used to generate a small-world network, with parameter *p* indicating the randomness of the network and *k* indicating the average number of neighours of agents. The Barabasi-Albert model^34^ is used to generate a scale-free network, with an initial population of 5 agents and a new agent with 2 edges added to the network at every time step. The results in Fig. 1 show that the three adaptive learning approaches under the proposed model outperform the static learning approach in all three networks in terms of a higher level of consensus and a faster convergence speed (except that SLR performs as well as SL in the scale-free network). Through dynamically adapting their learning behaviours during the opinion formation process, agents are able to reach an agreement more easily using the proposed adaptive learning approaches. In all networks, approach SBR is the most efficient approach, followed by SER and then SLR. This pattern of results demonstrates that a consensus can be further facilitated when agents adapt their learning rate and exploration rate simultaneously. The bottom row of Fig. 1 shows the dynamics of the agents’ opinions using adaptive learning approach SBR in the three networks. As can be seen, initially, the four opinions are adopted by the agents equally. As interactions proceed, the proportions of three opinions decrease gradually and one remaining opinion emerges as the consensus of the agents. It can also be observed that the different kinds of networks can produce various dynamics of consensus formation using the four learning approaches. Clearly, the scale-free network is the most efficient network for achieving high level of consensus compared with the other two networks. Previous studies have shown that this effect is due to the small graph diameter of scale-free networks^11^,^39^,^40^,^41^.

**Figure 2:**
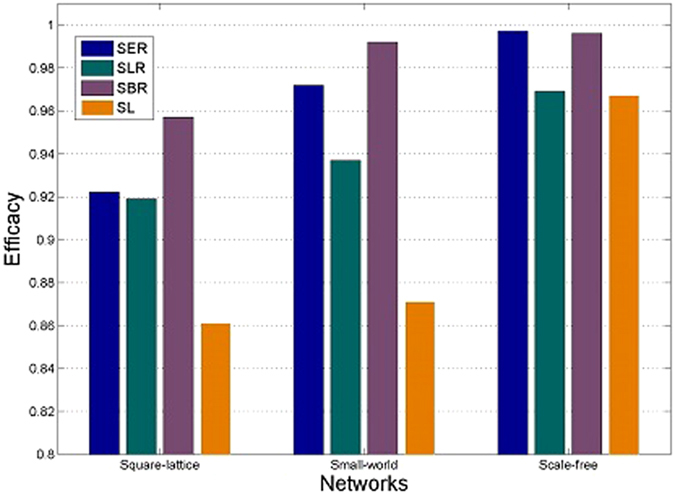
Efficacy of the four learning approaches in different kinds of networks. The parameter settings are the same as in Fig. 1.

Figure 2 plots the comparison of efficacy (i.e., the average ratio of agents in the population that can achieve the consensus) of the four learning approaches in three different networks. The three adaptive learning approaches outperform the static learning approach in all three networks. For example, in square-lattice network, SL can only enable averagely 86.1% agents in the population to achieve a consensus. This performance is upgraded to as high as 92.2%, 91.9% and 95.7% using the three adaptive learning approaches, respectively. The scale-free network can bring about the highest level of consensus among the three networks, confirming that scale-free network is the most efficient network for forming consensus. Note that in scale-free networks, the efficacy of SER and SBR is a little below 1 due to the exploration process in these two approaches.

Table 1 summarizes the final performance of the different approaches in 10000 independent runs. In order to better demonstrate the different performance of these approaches, we also include the results when 100% agents have achieved the final consensus. Achieving 100% level of consensus is an extremely challenging issue due to the widely recognized existence of subnorms formed in difference areas in the network. Clearly, the adaptive learning approaches outperform the static learning approach in all aspect of comparison. For example, in the square-lattice network, the possibility that a norm can successfully emerge (i.e., effectiveness) using SL is quite low (i.e., 55.0% for 90% convergence, and 46.6% for 100% convergence). The adaptive learning approaches, however, can greatly increase the possibility of norm emergence (e.g., 86.7% for 90% and 100% convergence using SBR). As for efficiency, it takes averagely 4288 steps for 100% convergence using SL, against 4113, 1180 and 1029 steps using the three adaptive learning approaches, respectively. To sum up, the adaptive learning approaches can achieve more robust formation of consensus among agents with fewer steps, compared with the static learning approach. The same pattern of results can also be observed in the small-world network and the scale-free network. The only difference is that SL can already perform very well in the scale-free network. The proposed three approaches, however, can further increase the performance to nearly 100% convergence in two different convergence levels.

**Figure 3:**
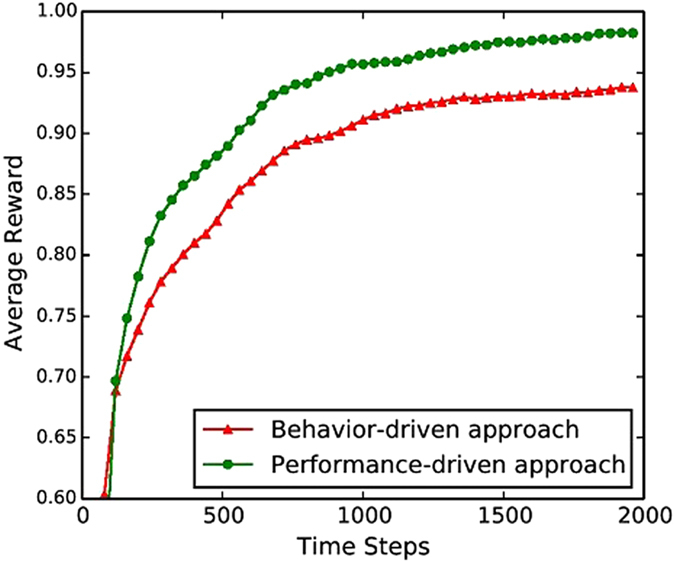
Comparison of the two different approaches to generate a guiding opinion in the model. The network topology is a small-world network, with *p* = 0.1 and *K* = 12. Other parameter settings are the same as in Fig. 1.

The performance of the two different kinds of approaches to generate a guiding opinion is shown in Fig. 3. As can be seen, the performance-driven approach outperforms the behaviour-driven approach in terms of a higher level of convergence and a faster convergence speed. This result implies that it is more reasonable to use the most profitable opinion rather than the most adopted opinion in the past as the competing strategy in EGT. This is due to the fact that agents are aiming at maximizing their own rewards. If an opinion has brought about the highest reward among all the opinions in the past, this opinion is the most profitable one and thus can be more likely to be imitated by the others. Dissemination of this kind of profit opinions will increase the consistency of agents’ opinions, which will further increase the performance of these opinions. Thus, the consensus formation process can be promoted accordingly.

**Figure 4:**
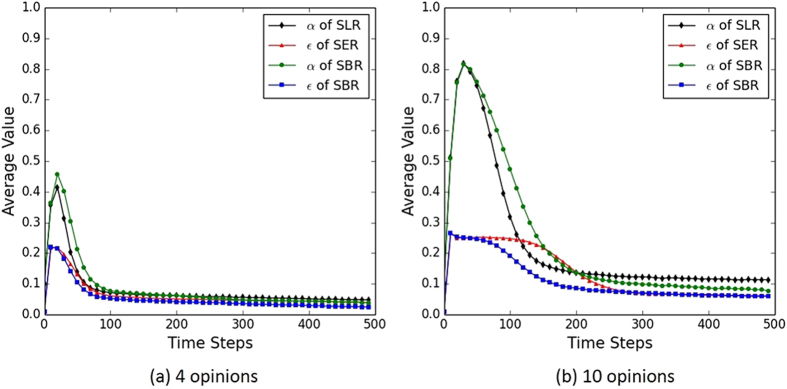
Dynamics of *ε* and *α* using the proposed learning approaches. The network topology is a small-world network with 100 agents, each having averagely 12 neighbours. Other parameter settings are the same as in Fig. 1.

To have a better understanding of the dynamics under the proposed model, it is necessary to see how the critical learning parameters of learning rate and exploration rate evolve during the process of consensus formation. The dynamics of *ε* and *α* using the proposed learning approaches with different sizes of opinion space are shown in Fig. 4. In both cases of opinion space, the values of *α* and *ε* increase sharply at the beginning, and then drop gradually to nearly zero. This is because the whole agent system is still in chaos at the beginning of learning as agents are not sure which opinion is the best and thus express their opinions randomly. In this case, it is more likely that the agents are in a “losing” state caused by failed interactions among the agents. In order to get over the “losing” state, agents would increase their learning rate and/or exploration rate to learn faster and/or explore more from the interactions. As the process moves on, each agent’s opinion choice is more and more consistent with its guiding opinion. Thus, *ε* and *α* decrease accordingly to indicate a “winning” state of the agents. The difference between Fig. 4(a,b) indicates that, in 10-opinion scenario, the values change more drastically at first and then it takes a longer time for these values to decrease to zero. This is because agents are more likely to choose the same opinion for achieving a consensus in a smaller size of opinion space. When the number of opinions gets larger, the probability to find the right opinion as the consensus is greatly reduced. The large number of conflicts among the agents thus cause the agents to be in a “losing” state more often in a larger opinion space, and thus the consensus formation process is greatly prolonged.

**Figure 5:**
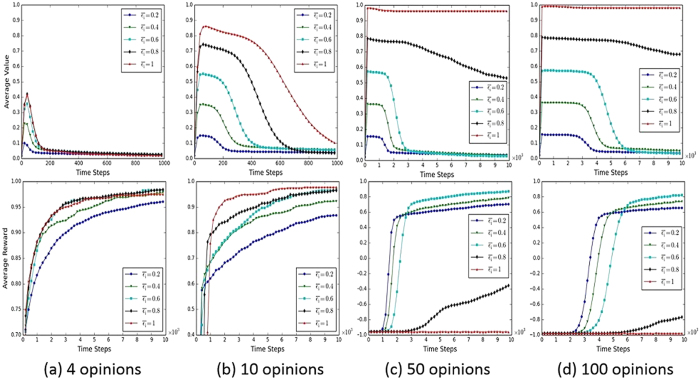
Dynamics of *ε* and consensus formation with varying 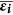 in different sizes of opinion space. The top are the dynamics of *ε* in four cases of opinion space, and the bottom are the corresponding learning dynamics of consensus formation in each case. Parameter settings are the same as in Fig. 1.

Parameter 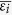 is a crucial factor in affecting the dynamics of consensus formation using SER and SBR, due to its functionality of confining the exploration rate to a predefined maximal value. It can be expected that, with different sizes of opinion space, different values of 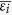 may have diverse impacts on the learning dynamics as agents can have different numbers of opinions to explore during learning. Figure 5 shows the dynamics of *ε* and corresponding learning curves of consensus formation using SER when 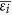 is chosen from a set of {0.2, 0.4, 0.6, 0.8, 1}. Four cases are considered to indicate different sizes of opinion space, from small size of 4 opinions to large size of 100 opinions. In case of 4 opinions, the dynamics of *ε* share the same patterns under different values of 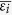. The values spike sharply at the beginning process of learning, and then drop gradually to zero. The peaks of *ε*, however, differ from each other, from around 0.1 when 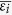 = 0.2 to around 4.4 when 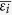 = 1. This is because a larger 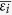 enables the agents to explore more opinion choices during learning. Higher exploration accordingly causes more failed interactions among the agents, and thus the exploration rate *ε* will increase further to indicate a “losing” state of the agent. The corresponding learning curves in terms of average rewards of agents indicate that the consensus formation process is hindered when using a small value of 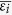. The same pattern of dynamics can be observed when the agents have 10 opinions. The only difference is that the peak values are higher than those in case of 4 opinions, and it takes a longer time for these values to decline to zero. The dynamics patterns, however, are quite different in cases of 50 and 100 opinions. In these two scenarios of large size of opinion space, the values of *ε* cannot converge to zero when 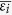 = 1 and 0.8 in 10^4^ time steps. This is because agents have a large number of alternatives to explore during the learning process, which can cause the agents to be in a state of “losing” consistently. This accordingly increases the values of *ε* until reaching the maximal values of 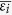. As a result, a consensus cannot be achieved among the agents, which can also be observed from the low level of average rewards at the bottom low of Fig. 5(c,d). Although *ε* can gradually decline to zero when 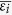 = 0.6, 0.4, and 0.2, the dynamics of consensus formation in these three cases vary a bit. The consensus formation processes are slower at first when 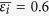, but then catch up with those when 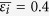 and 0.2, and then keep faster afterwards. The general results revealed in Fig. 5 can be summarized as follows: (1) in a relatively small size of opinion space (e.g., 4 opinions and 10 opinions), the values of *ε* under various 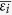 can converge to zero after reaching the maximal points, and a larger 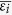 in this case can bring about a more efficient process of consensus formation among the agents; and (2) when the size of opinion space becomes larger (e.g., 50 opinions and 100 opinions), a higher value of 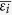 can greatly hinder the process of consensus formation. A tipping point of 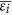 exists between promoting the consensus formation and prolonging it.

**Figure 6:**
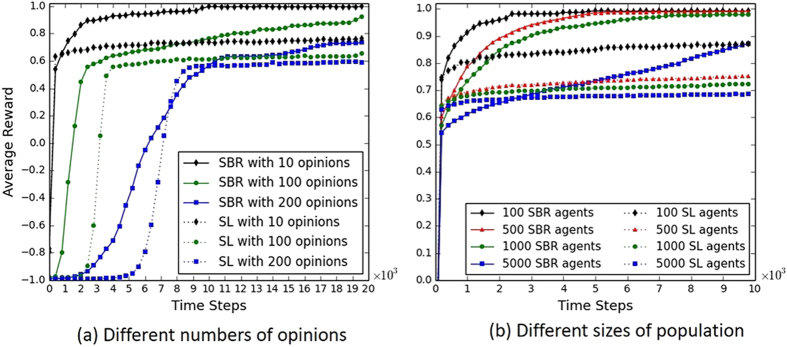
Influence of sizes of opinion space (**a**) and population (**b**) on dynamics of consensus formation in small-world networks, comparing adaptive learning approach SBR with static learning approach SL. In the small-world networks, *p* = 0.1 and *K* = 12. In (**a**), the population size is 100, and in (**b**), the size of opinion space is 4. Other parameters are set to the default values as in Fig. 1.

The results between SL and the adaptive learning approach SBR with different sizes of opinion space is given by Fig. 6(a). It can be seen that a larger number of available opinions results in a delayed convergence of consensus among the agents. This is because a larger number of opinions are more likely to produce local clusters of conflicting opinions (i.e., sub-norms), leading to diversity across the population. It thus takes a longer time for the agents to eliminate this diversity and achieve a global consensus, and accordingly the process of consensus formation is prolonged throughout the network. In all cases, the adaptive learning approach SBR performs better than approach SL in terms of a faster convergence speed and a higher convergence level. In situations of 100 and 200 opinions, the consensus formation process is still converging after 10000 steps when using SBR. This result shows that the proposed adaptive learning model is indeed effective for achieving consensus in a large opinion space. The influence of population size on dynamics of consensus formation is shown in Fig. 6(b). In both approaches of SL and SBR, the convergence process is hindered as the population is growing larger. This result occurs because the larger the society, the more difficult to diffuse the effect of local learning to the whole society. This phenomenon can be observed in human societies where small groups can more easily establish social norms than larger groups^31^. The proposed adaptive learning approach SBR, however, can greatly facilitate consensus formation in different population sizes. In cases of 100, 500 and 1000 population size, SBR can achieve almost 100% convergence, which is a great promotion from the low convergence levels using SL. In a population of 5000 agents, the consensus formation process is steadily facilitated to a level of 90% during 10000 steps using SBR, against a convergence level close to 70% using SL.

**Figure 7:**
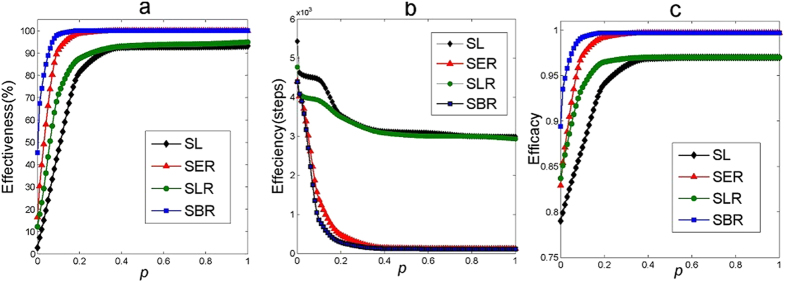
Influence of network randomness on consensus formation (100% convergence) in small-world networks. The rewiring possibility *p* is a parameter in the Watts-Strogatz model^33^ to indicate different levels of network randomness. When *p* = 0, the network is reduced to a regular ring lattice. Increasing rewiring probability *p* produces a network with increasing randomness. When *p* = 1, the network becomes a fully random network. The network population is 100 with each agent having averagely 12 neighbours (i.e., *K* = 12). Other parameter settings are the same as in Fig. 1.

Figure 7 presents the performance of 100% consensus formation (i.e., all the agents reaching a consensus) using the four learning approaches in small-world networks with various randomness. As can be seen, it is more efficient for a consensus to emerge in a network with higher randomness. This is because increasing randomness can reduce the network diameter (i.e., the largest number of hops in order to traverse from one vertex to another^37^), and it is more efficient for a network to achieve a consensus in a network with smaller diameter^11^. The results also show that a minor increase of rewiring possibility *p* from 0 to 0.1, especially from 0.01 to 0.1, can bring about significant improvement of consensus formation, while further increasing the rewiring possibility from 0.2 to 1.0 cannot cause a further significant improvement. This is due to the fact that the network randomness is already quite high when the rewiring possibility *p* is in-between [0.01, 0.1]. In all scenarios, the proposed learning approaches outperform the static learning approach in all three comparison criteria. Specially, when the randomness is high, approach SER and SBR can achieve a consensus with 100% possibility. This robust norm emergence, however, only takes very short converging time (e.g., 117 and 112 steps for SER and SBR, respectively, compared with 2984 steps for SL, when *p* = 1.0.).

**Figure 8:**
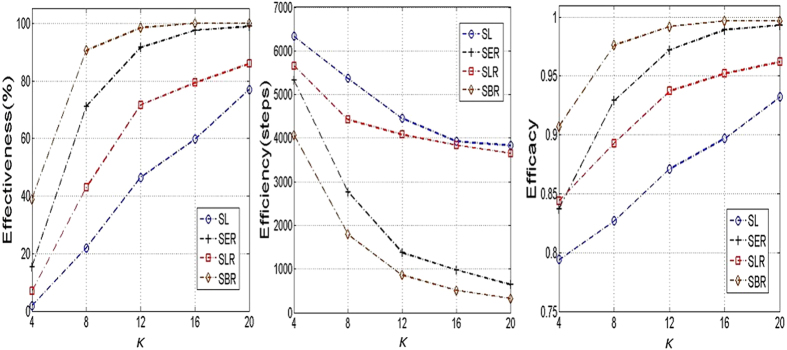
Influence of number of neighbours on consensus formation (100% convergence) in small-world networks. The network population is 100 and rewiring probability *p* is 0.1. Other parameter settings are the same as in Fig. 1.

Figure 8 shows the influence of number of neighbours *K* on consensus formation in small-world networks. The results imply that, in all scenarios, consensus formation is steadily promoted when the average number of neighbors increases. This effect is due to the clustering coefficient of the network, which is a measure of degree to which nodes in a graph tend to cluster together^42^. When the average number of neighbors increases, the clustering coefficient also increases. Therefore, agents located in different parts of the network only need a smaller number of interactions to reach a consensus. On the other hand, when agents have a smaller neighborhood size, they only interact with their fewer neighbors, which account for a smaller proportion of the whole population. This results in clusters of diverse opinions formed at different regions of the network. Such contradictory opinions conflict with each other in the network, and thus more interactions are needed to solve these conflicts and achieve a uniform consensus for the whole society. In all cases of neighborhood sizes, the three adaptive learning approaches can bring about more robust formation of consensus with a faster convergence speed and a higher convergence level than the static learning approach. As for effectiveness, the percentage of runs in which all the agents can achieve a consensus using SL is 1.8%, 22%, 46.5%, 59.8%, 77.0%, when *K* = {4, 8, 12, 16, 20}, respectively. The three adaptive learning approaches, however, can greatly increase the likelihood of consensus formation (e.g. {38.9%, 90.6%, 98.4%, 100%, 100%} for corresponding neighbourhood size using SBR). With the increase of *K*, the steps needed for achieving a consensus are reduced (from 6336 steps to 3832 when *K* increases from 4 to 20). In each case of neighbourhood size, the adaptive learning approaches require fewer steps for achieving a consensus than SL. The improvement is more distinct using SBR and SER when *K* becomes larger. For example, when *K* = 20, it only takes 325 steps to achieve a consensus using SBR, which is against 3832 steps using SL. This demonstrates the benefits of adapting learning, especially adapting exploration rates, in boosting the efficiency of consensus formation. As for efficacy, the proportion of agents achieving the same consensus is {0.794, 0.827, 0.871, 0.897, 0.932} using SL, respectively. This level of consensus can be increased to {0.907, 0.976, 0.992, 0.997, 0.997} respectively using SBR, which implies that a much higher level of consensus can be achieved using the adaptive learning approaches.

**Figure 9:**
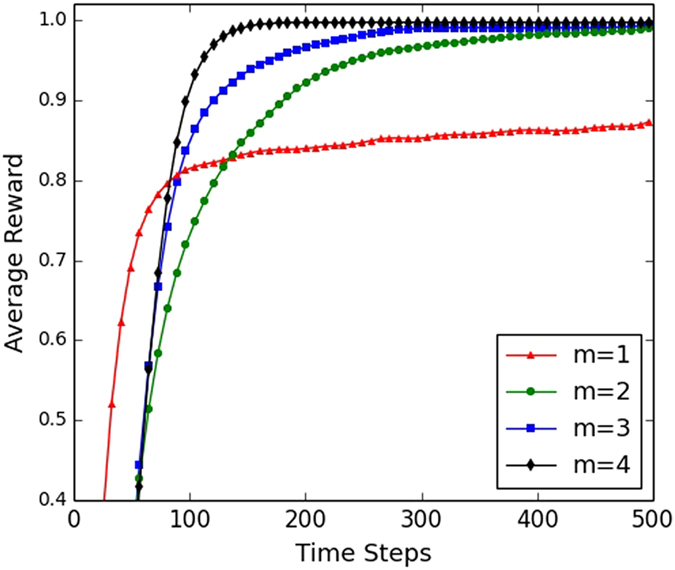
Influence of number of neighbours on consensus formation in scale-free networks. The scale-free networks are generated according to the Barabasi-Albert model, starting from 5 nodes and a new node with *m* = 2 edges connected to an existing node at each step. This will yield a network with an average degree of 2*m*. The figure plots how the parameter of *m* affects the consensus formation process using adaptive learning approach SER in a network population of 100 agents.

We have also investigated how the average number of neighbours affects consensus formation in scale-free networks. The general result pattern is similar to that in small-world networks, i.e., the increase of average number of agents can boost the consensus formation among agents. As an example, Fig. 9 plots the dynamics of consensus formation against the average number of neighbours in terms of parameter *m* (i.e., the number of edges connected to an existing node at each step in the Barabasi-Albert model) using adaptive learning approach SER. The result shows that as the average number of neighbours increases, the consensus formation process is greatly facilitated. In more detail, when *m* = 1, the effectiveness is 3%, which means that there are only 3% percentage of runs in which a 100% consensus can be achieved, and this consensus takes an average of 6032 steps to be established. When *m* is increased to 2, 3, 4, the effectiveness is greatly upgraded to 100%. This robust consensus formation, however, only takes an average of 228, 128, 112 steps, respectively.

## Discussion

In general, two exclusive research paradigms, i.e., individual learning versus social learning, coexist in the literature for studying opinion dynamics in social networks, focusing on different perspectives of agent learning behaviours. The “individual learning” perspective considers that an agent learns from trail-and-error interactions solely based on its individual experience^31^, while the “social learning” perspective enables individuals to obtain information and update their beliefs and opinions as a result of their own experiences, their observations of others’ actions and experiences, as well as the communication with others about their beliefs and behavior^24^,^43^. In this sense, the broad literature in statistics, especially statistical physics and social physics, has studied dynamics and evolution of opinions from a social learning perspective, focusing on macroscopic phenomenon achieved through local dynamics that are based on simple social learning rules, such as local majority or imitating a neighbor^7^,^20^,^25^. Social learning can be conducted through either a Bayesian or a non-Bayaeian learning process, depending on whether agents update their opinions or beliefs given an underlying model of the problem^24^.

On the other hand, there is abundant work in the multiagent systems (MASs) community to investigate consensus formation from individual learning perspective^12^,^31^,^44^. In this area, consensus is usually termed as *social norm*, and the process of consensus formation is thus alternated by the phrase of *emergence of social norms*. The focus of studies in this area is to examine general mechanisms behind efficient consensus formation (i.e., norm emergence) while agents interact with each other using basic individual learning (particularly RL) methods. For example, Sen *et al.*^31^,^45^ proposed a framework for the emergence of social norms through random learning based on private local interactions. This work is significant because it indicates that agents’ private random learning is sufficient for emergence of social norms in a well-mixed agent population; Villatoro *et al.*^12^,^37^,^42^ investigated the effects of memory of past activities during learning on the emergence of social norms in different network structures, and used two social instruments to facilitate norm emergence in networked agent societies; More recently, authors in^28^,^44^,^46^ proposed a collective learning framework for norm emergence in social networks in order to model the collective decision making process in humans. Although these studies provide valuable insights into understanding efficient mechanisms of consensus formation, they share the same limitation to answer a critical question, that is, how can agent learning behaviours directly influence the process of consensus formation? In other words, learning parameters in these studies are often fine-tuned by hand and thus cannot be adapted dynamically during the process of consensus formation. This assumption is against the essence of human decision making in real-life, when people can dynamically adapt their learning behaviours during interaction and exchange of their opinions, rather than simply follow a fixed learning schedule. Our work, thus, takes a different perspective from the above studies by investigating the impact of adaptive behaviours during learning on consensus formation. The main conclusion is that apart from various previous reported mechanisms such as collective interaction protocols and utilization of topological knowledge, learning itself can play a vital role in facilitating consensus formation among agents.

The highlight of the proposed model in this paper is the integration of social learning into the local individual learning in order to dynamically adapt agents’ learning behaviours for a better performance of consensus formation. Our work thus bridges the gap between the two distinct research paradigms for opinion dynamics by coupling a social learning process (through imitation in EGT) with a local individual learning process (i.e., RL). Although it can be expected that requiring communication among agents or additional information through social learning can facilitate formation of consensus, this is not straightforward in the proposed model as the synthesised information used in social learning is generated from trail-and-error individual learning interactions, and this information is then utilized as a guide to heuristically adapt the local learning further. Tight coupling between these two learning processes can make the whole learning system rather dynamic. However, by synthesising the individual learning experience into competing strategies in EGT and adapting local learning behaviours based on the principle of “Win-or-Learn-Fast”, our work has illustrated that this kind of interplay between individual learning and social learning is indeed helpful in facilitating the formation of consensus among agents.

The long term goal of this research is to gain a deeper understanding of the role of individual learning and social learning in facilitating consensus formation in social networks. Although we only focus on EGT as the social learning strategy and Q-learning as the individual learning strategy in this paper, there are various kinds of individual learning as well as social learning strategies in the literature. For example, social learning can be conducted as a majority voting process, a strategy diffusion process^47^,^48^, an epidemics infection process^49^, or a crowd herding process^7^. It thus would be interesting to test the proposed framework using other types of learning strategies in the model in order to analyze their influence on the dynamics of opinions. Moreover, although the model proposed in this paper is just a theoretical one, the idea of coupling an individual learning process with a social learning process in the evolution process of opinions would provide some useful insights into experimental investigations of human’s adaptive behaviours in real scenarios. Such insights could thus be helpful to interpret fundamental mechanisms of consensus formation in human societies.

In the model, two main challenging technical issues are: (1) how to generate guiding opinions simply based on agents’ own historical learning experience? and (2) how to adapt agents’ local learning behaviors based on the generated guiding opinions? To solve the former problem, the historical learning experience of each agent is synthesised into a strategy that competes with other strategies in the population based on the principle of EGT. The strategies that have better performance are more likely to survive and thus be accepted by other agents. For the latter, the concept of “winning” or “losing” in the well-known Multi-Agent Learning (MAL) algorithm WoLF (Win-or-Learn-Fast)^38^ is elegantly borrowed to indicate whether an agent’s behavior is consistent with the guiding opinion. According to the “winning” or “losing” situation, agents then can dynamically adapt their learning behaviors in local layer learning. It should be noted that the WoLF heuristic applied in the model is a quite general mechanism that has been widely used in different forms by previous studies. For example, in the study^50^, the winning or losing concept is analogous to whether the strategy of a player is the same as that of the majority of other players. If the player’s strategy is the same as that of the majority of its neighbours, the player is considered to be in a winning state and thus its learning activity will be low. Conversely, if the strategy is different from that of the majority (i.e., it is losing), the learning activity of the player will be high. It has been shown that this kind of simple heuristic is effective for achieving consensus of cooperation in social dilemmas. Another example is the well-known “win-stay, lose-shift” (WSLS) strategy^51^, which has also been shown to be an effective mechanism for solving cooperation problems in social dilemmas. Using WSLS, an agent repeats the previous move if the resulting payoff has met its aspiration level and changes otherwise. Although the WoLF heuristic in our model is realized in a different way from the the above models, the main principle embodied in them is quite similar, namely, an agent should act (e.g., learn, copy or transform it behaviours) slowly when it is performing well and fast otherwise. We therefore expect the WoLF principle to be a general and effective mechanism for modelling human’s adaptive behaivours in resolving conflicts in human societies. Further empirical investigations are needed to verify this hypothesis as this could lead to new interesting results in both behavioral economics and social sciences.

## Additional Information

**How to cite this article**: Yu, C. *et al.* Modelling Adaptive Learning Behaviours for Consensus Formation in Human Societies. *Sci. Rep.* **6**, 27626; doi: 10.1038/srep27626 (2016).

## Figures and Tables

**Table 1:** Comparison of *Effectiveness* and *Efficiency* in the three networks using the four learning approaches.

Square-lattice	*C*_90%_		*C*_100%_	
	Effectiveness	Efficiency	Effectiveness	Efficiency
SER	74.7%	1087	74.7%	1180
SLR	74.8%	1509	66.1%	4113
SBR	86.7%	970	86.7%	1029
SL	55.0%	1617	46.6%	4288
Small-world	90% convergence		100% convergence	
	Effectiveness	Efficiency	Effectiveness	Efficiency
SER	91.7%	1692	91.6%	1735
SLR	84.2%	1969	71.6%	4077
SBR	98.4%	818	98.4%	862
SL	54.9%	2212	46.5%	4450
Scale-free	90% convergence		100% convergence	
	Effectiveness	Efficiency	Effectiveness	Efficiency
SER	100%	181	100%	246
SLR	99.9%	183	93.1%	3075
SBR	100%	114	100%	162
SL	99.1%	331	90.4%	3204
